# Exploratory secondary analysis of published aggregate growth and metabolic data in children born small for gestational age treated with growth hormone

**DOI:** 10.3389/fendo.2026.1942946

**Published:** 2026-09-25

**Authors:** Huihua Yuan, Yanli Wang, Zhijin Lu, Zhigang Zhang, Shuyi Luo, Yan Wang

**Affiliations:** Department of Pediatric Endocrinology, Dongguan Maternal and Child Health Hospital, Dongguan, China

**Keywords:** aggregate data analysis, ecological inference, growth hormone, metabolic surveillance, registry research, secondary analysis, small for gestational age, somatropin

## Abstract

**Purpose:**

To describe published annual growth and metabolic summaries for children born small for gestational age (SGA) treated with growth hormone and to define the limits of inference from aggregate registry data.

**Methods:**

We conducted an exploratory secondary analysis of published aggregate means, standard deviations, and outcome-specific sample sizes from baseline through year 5 in the SGA subgroup of the Korean LG Growth Study, with up to 318 children represented at baseline. Primary reporting was descriptive and included annual values, changes from baseline, and measurement availability. Exploratory slope models were retained only as supplementary sensitivity analyses. The published aggregate LGS summaries available for the present secondary analysis did not include longitudinal blood-pressure measurements or cardiovascular-event records.

**Results:**

Height SDS was −2.38 at baseline and −0.76 at year 5, weight SDS was −2.21 and −0.84, and BMI SDS was −0.93 and −0.64. The published mean AST was 32.07 IU/L at baseline and 24.28 IU/L at year 5, whereas several other metabolic markers had higher year-5 means. Median year-5 measurement availability was 12.8% of outcome-specific baseline counts, and only six triglyceride measurements were available at year 5. The markedly reduced outcome-specific sample sizes at later follow-up points substantially limit interpretation of long-term patterns.

**Conclusion:**

Published annual mean values differed across follow-up, but changing outcome-specific samples and the unavailability of individual-level longitudinal data to the present secondary analysis preclude estimation of within-child change, causal treatment effects, or cardiovascular risk.

## 1 Introduction

Small for gestational age is a birth-size classification rather than a single disease. International guidance defines SGA using birth weight and/or length below -2 standard deviations for gestational age and sex, followed by clinical attention to the cause of impaired fetal growth and the pattern of postnatal recovery (1). Most children born SGA show catch-up growth during the first two years of life, whereas a smaller proportion remains short after the period in which spontaneous recovery is expected. These children require a diagnostic evaluation to distinguish persistent SGA-related short stature from syndromic, skeletal, endocrine, nutritional, and chronic systemic causes. The therapeutic context is thus not solely based on height, but on a life-course framework (2, 3). Only a minority of children born SGA remain sufficiently short to meet clinical criteria for GH evaluation, as illustrated by prevalence data from Italy (4).

Recombinant human GH is a proven therapy for persistently short children born SGA. The goal is not to normalize all measurements, but to optimize the height velocity, minimize the difference from the target height, and maximize the chance of reaching the adult height within the population range. Improvement in height SDS during growth hormone treatment has been reported across cohorts in Europe, Japan, Korea, and China, with earlier treatment initiation and sustained adherence generally associated with more favorable growth outcomes (5–9). Recent research also indicates age at onset, baseline height, treatment duration, dosage, genetic or epigenetic results, prematurity and GH deficiency as factors affecting response (10–13).

Metabolic monitoring remains important alongside the expected auxological response to growth hormone therapy. GH promotes lipolysis and lean-tissue accretion and interacts with the insulin-like growth factor system; during active treatment, it may also reduce peripheral insulin sensitivity. In children born SGA, this pharmacologic effect occurs against a background of developmental and familial factors that may also influence adiposity, glucose regulation, lipid metabolism, and blood pressure. Therefore, an increase in insulin, glucose or HbA1c levels during therapy should not be interpreted as evidence of toxicity, but should not be discounted because of mean levels that are still within a laboratory reference range. The relevant questions are whether change is sustained, clinically meaningful, concentrated in vulnerable subgroups, or reversed after treatment stops (1, 14).

Recent SGA cohorts provide broadly reassuring but not uniform evidence. Ten-year observational follow-up in Spain found an early increase in HOMA-IR that stabilized within the normal range, while height continued to improve (15). The GH-RAST study reported increases in glucose, insulin, and triglycerides that remained clinically acceptable, alongside decreases in total and LDL cholesterol (16). A smaller Italian cohort found sustained growth with no overt hyperglycemia or dyslipidemia, although glycemia warranted continued surveillance (17). The five-year LG observational analysis reported maintained random glucose within the normal range, declining total cholesterol, and improvements in several growth and liver-related measures (18). These studies agree on efficacy but differ in follow-up duration, laboratory availability, fasting status, pubertal composition, and analytical handling of repeated observations.

Cardiovascular safety is more difficult to resolve than metabolic biomarker change. A Swedish population-based cohort found a higher relative risk of cardiovascular events among individuals treated with GH in childhood, including the SGA subgroup, although absolute event rates were low and residual confounding could not be excluded (19). The multinational SAGhE cohort reported diagnosis-specific mortality patterns and an increased standardized mortality ratio in SGA, but the result was driven largely by one national subcohort and did not show a consistent dose-response relation (20). By contrast, a Dutch study that followed previously treated SGA adults for 12 years after cessation found metabolic and blood-pressure profiles similar to untreated SGA and appropriate-for-gestational-age controls, apart from characteristics shared by SGA groups (21). Brain MRI follow-up likewise found no increased prevalence of selected cerebrovascular abnormalities attributable to treatment, while acknowledging low event counts (22).

Large real-world safety programs have improved the evidence base but have not eliminated the central identification problem. KIGS, NordiNet IOS, ANSWER, SAGhE, PATRO, and national data linkages include tens of thousands of treated children and permit detection of uncommon events that randomized trials cannot address (23–25). However, these programs have a variety of different diagnoses, doses, treatment periods, lab schedules and background risks. Treated children are not directly comparable with the general population, and untreated SGA comparator groups remain uncommon. Attrition is particularly important because continued follow-up may preferentially include children with greater disease severity, greater clinical concern, better access to specialist care, or better treatment adherence. The direction of bias cannot be determined by the number of observations that are missing.

The methodological gap addressed here is narrower than a claim of causal long-term safety. The published aggregate LGS summaries used for the present secondary analysis provide outcome-specific annual means, standard deviations, and sample sizes over five years; however, the materials available to us did not include individual-level trajectories, a common identifiable cohort across years, an untreated comparison group, person-time, or cardiovascular-event records. These summaries can describe differences among published annual group means and make late measurement sparsity transparent, but they cannot support within-child longitudinal modeling, causal treatment-effect estimation, incidence rates, or cardiovascular-risk estimates. Accordingly, the present study treats the annual summaries primarily as descriptive aggregate data; exploratory slope analyses are confined to the **Supplementary Material**.

Although the analyzed cohort is Korean, the data-quality challenges are relevant to emerging Chinese growth-hormone registries, particularly outcome-specific missingness and incomplete post-treatment follow-up (9, 26–28). The Korean estimates are not interpreted as outcomes for Chinese patients. This study had two objectives. First, it described the published annual growth and metabolic means and outcome-specific measurement availability from baseline through year 5. Second, it evaluated how declining and potentially changing annual samples constrain interpretation of these published summaries. The research questions were: (RQ1) What patterns are visible in the published annual growth and metabolic means? (RQ2) How does outcome-specific measurement availability affect the interpretation of later annual summaries?

The contribution of this study is primarily methodological and interpretive rather than the identification of new patient-level treatment effects. By placing the published annual SGA summaries within a single outcome-by-year framework, the analysis makes the extent of outcome-specific measurement loss explicit and distinguishes descriptive changes in published annual means from within-child longitudinal change. The study therefore clarifies what can and cannot be inferred from published aggregate registry summaries and identifies where patient-level data would be required for valid treatment-effect or long-term safety analyses.

## 2 Methodology and data analysis strategy

### 2.1 Research design

This study was an exploratory secondary analysis of published annual aggregate summaries from the SGA subgroup of the LG Growth Study. The analytical unit was the annual outcome summary rather than the individual child. Published means, standard deviations, and outcome-specific sample sizes were available from baseline through year 5. The main analysis described annual values and measurement availability; it did not estimate individual longitudinal or causal treatment effects.

### 2.2 Data source, population, time period, and scope

The analyzed values were transcribed from the published SGA-subgroup tables reported by S. Kim et al. (12), which were derived from the LG Growth Study, a multicenter Korean observational registry. The source article described up to 318 children in the SGA subgroup at baseline; however, the present analysis did not use individual participant records. The authors did not have access to the original individual-level LGS database. Therefore, throughout this manuscript, statements that information was unavailable refer to the published aggregate summaries used for the present secondary analysis and should not be interpreted as evidence that the corresponding variables or longitudinal information were not collected in the underlying LGS registry. At GH initiation, the source article reported a mean age of 6.5 years (SD 2.4), 171 male participants (53.8%), and a mean treatment duration of 3.8 years (SD 1.3). Baseline aggregate means were −2.38 (SD 0.63) for height SDS, −2.21 (SD 0.99) for weight SDS, and −0.93 (SD 1.04) for BMI SDS. Annual aggregate values were available from baseline through year 5, with outcome-specific sample sizes that varied across measures and follow-up years. Two authors independently checked each outcome-year mean, standard deviation, and sample size against the corresponding published source table. Any discrepancy was resolved by joint review of the original publication before the analytical dataset was finalized.

The geographic scope of the analyzed cohort is Korea. China is the policy and implementation context for interpretation because Chinese SGA-specific studies, treatment-pattern databases, and long-term registry plans are developing rapidly (9, 26–28). No result is presented as a Chinese patient estimate. The analysis period is defined by registry follow-up year rather than calendar year because the public table does not provide a common calendar entry date for each participant.

### 2.3 Available outcomes and data limitations

**Table 1** summarizes the outcomes available in the published aggregate data used for the present secondary analysis. Follow-up year indexes the annual published summaries, while the outcomes comprise growth, adiposity, liver-related, lipid, and glycemic measures. Participant-level covariation was not available in these published summaries; therefore, individual-level exposure, mediation, moderation, and covariate-adjusted analyses could not be performed. Age, sex, puberty, baseline adiposity, gestational age, growth hormone dose, adherence, and center therefore could not be adjusted for in the present analysis. These are limitations of the data available for this secondary analysis and are not confirmed limitations of the underlying LGS registry.

**Table 1:** Outcomes and data limitations in the published LGS SGA aggregate summaries used for the present secondary analysis.

Domain	Outcome	Unit	Baseline N	Year-5 N	Measurement role	Limitation of available aggregate data
Growth	Height SDS	SDS	318	42	Primary auxological outcome	Annual means may represent changing participant subsets
Growth	Weight SDS	SDS	311	42	Growth and body-size outcome	Does not distinguish lean from fat mass
Adiposity	BMI SDS	SDS	311	42	Proxy for relative adiposity	Waist circumference and body composition not reported in the aggregate summaries analyzed here
Liver-related	AST	IU/L	307	39	Nonspecific biochemical marker	Influenced by age, muscle, illness, and laboratory factors
Liver-related	ALT	IU/L	307	39	Nonspecific biochemical marker	Imaging and liver-disease adjudication not reported in the aggregate summaries analyzed here
Metabolic	Uric acid	mg/dL	208	19	Metabolic and renal-risk marker	Renal function, diet, and threshold status not reported in the aggregate summaries analyzed here
Lipid	Total cholesterol	mg/dL	273	29	Lipid-risk marker	LDL and HDL longitudinal summaries not available in the aggregate data analyzed here
Lipid	Triglycerides	mg/dL	70	6	Lipid-risk marker	Very sparse late data; fasting status not reported in the aggregate summaries analyzed here
Glycemic	Glucose	mg/dL	296	38	Glycemic marker	Reported as random glucose; visit-specific fasting status not reported in the aggregate summaries analyzed here
Glycemic	HbA1c	%	77	29	Longer-term glycemic marker	Irregular outcome availability across years
Cardiovascular	Blood pressure	—	Not reported in analyzed summaries	Not reported in analyzed summaries	Direct cardiovascular-risk marker	Hypertension cannot be estimated from the analyzed aggregate summaries
Cardiovascular	Clinical cardiovascular events	—	Not reported in analyzed summaries	Not reported in analyzed summaries	Clinical safety outcome	Event dates, person-time, linkage, and adjudication were not available in the analyzed aggregate summaries

Baseline and year-5 N values represent the number of reported measurements for each outcome, not a common complete-case cohort. References to information being “not reported” or “not available” in this table refer only to the published aggregate summaries used for the present secondary analysis and do not establish that the corresponding information was absent from the original LGS registry.

The source publication described the reported glucose measure as random glucose; visit-specific fasting status was not available in the aggregate summaries. Accordingly, the glucose series is not interpreted as a standardized fasting-glucose trajectory.

### 2.4 Descriptive analysis and measurement availability

The primary analysis was descriptive. For each outcome, the published mean, standard deviation, and outcome-specific sample size were tabulated at baseline and at years 1–5. Absolute differences from baseline and differences between adjacent published annual means were calculated to describe the observed pattern without assuming a constant longitudinal rate of change. Measurement availability was summarized using the number of reported observations at each follow-up year and, for visualization, the percentage relative to the outcome-specific baseline count. These percentages represent measurement availability rather than participant retention because participant identities across annual summaries were not available in the published aggregate data used for this analysis.

Annual samples may overlap, and cross-year covariance could not be determined from the published aggregate summaries available for the present analysis. Formal longitudinal hypothesis testing was therefore not used as the basis for the main conclusions. Exploratory slope and sensitivity analyses are reported only in the **Supplementary Material** and are not interpreted as repeated-measures or causal inference.

### 2.5 Software, reproducibility, and quality checks

Data were processed in Python using pandas, NumPy, statsmodels, matplotlib, and openpyxl. The analysis workflow validated required columns, numeric data types, positive standard deviations, sample sizes greater than one, duplicate outcome-year combinations, and the presence of years 0–5 for each outcome. Primary outputs comprised the published annual summaries, changes from baseline and between adjacent years, outcome-specific measurement availability, and descriptive figures. Exploratory inverse-variance weighted slopes, unweighted models, early-period models, and leave-one-time-point-out analyses were generated only as supplementary sensitivity outputs.

### 2.6 Ethical and data-use considerations

Only publicly available aggregate data were analyzed; no individual-level or identifiable patient information was accessed. Ethics and consent procedures for the original LG Growth Study were the responsibility of the original investigators.

## 3 Results

### 3.1 Available observations and follow-up sparsity

The SGA registry subgroup included 318 children, but the number contributing to each outcome differed at baseline. Height SDS had 318 observations, weight and BMI SDS 311, AST and ALT 307, glucose 296, total cholesterol 273, uric acid 208, HbA1c 77, and triglyceride 70. By year 5, the corresponding counts were 42 for the three auxological outcomes, 39 for AST and ALT, 38 for glucose, 29 for total cholesterol and HbA1c, 19 for uric acid, and six for triglyceride.

Measurement availability therefore declined markedly and differed by outcome. These counts should not be interpreted as conventional cohort-retention rates because laboratory testing was outcome-specific and participant identities across annual summaries were not available in the published aggregate data used for this analysis. Late annual means were consequently calculated from substantially smaller and potentially clinically different subsets of patients. Selection bias may operate in either direction if continued testing was related to treatment response, clinical concern, adherence, treatment duration, or access to follow-up. The reasons for missing outcome measurements were not reported in the published aggregate summaries available for the present analysis; therefore, the mechanism or direction of any resulting selection cannot be established from these data. The year-5 uric-acid and triglyceride summaries are especially sparse and are not reliably interpretable for long-term trajectory claims. **Figure 1** displays outcome-specific measurement availability across follow-up.

**Figure 1:**
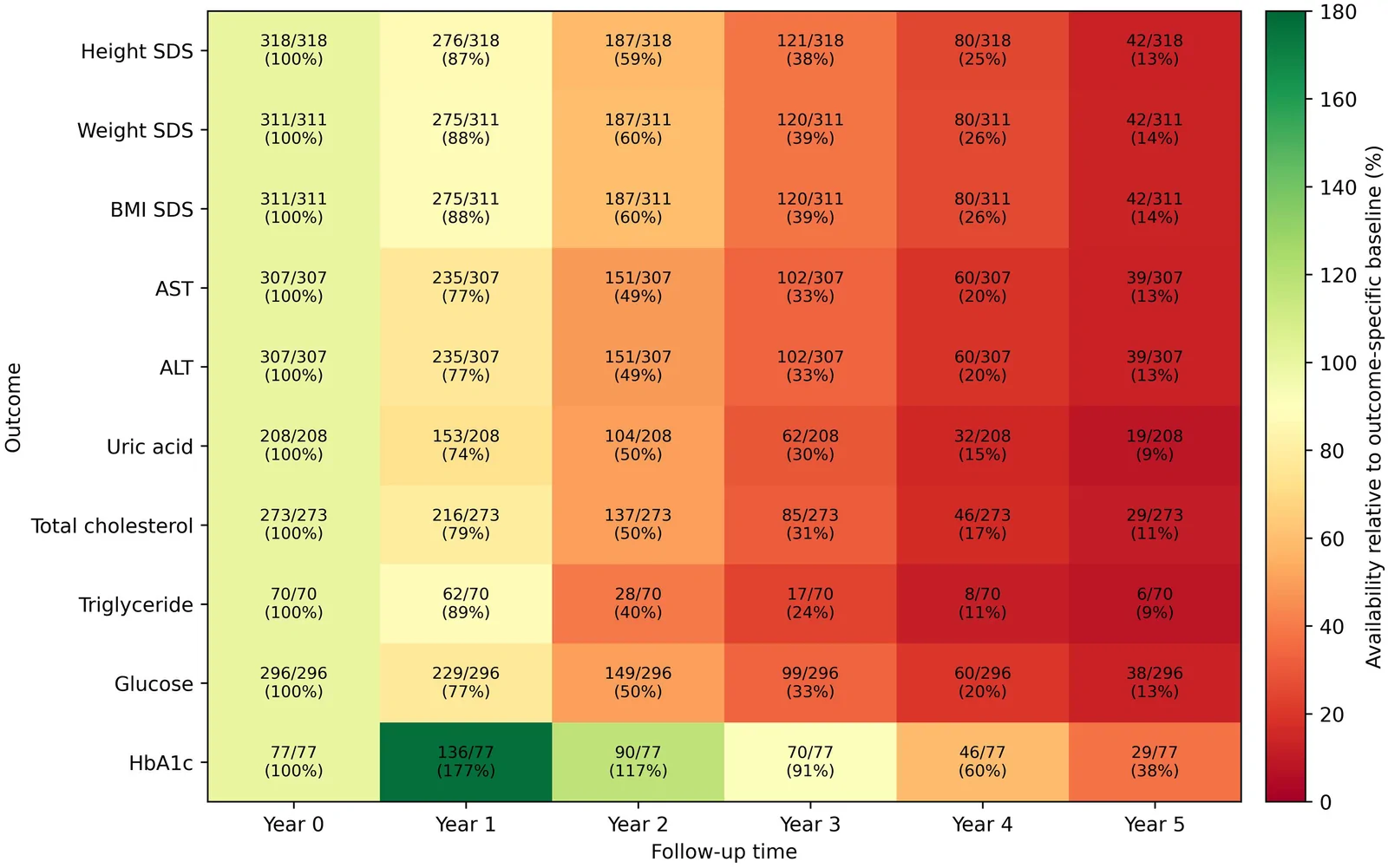
Outcome-specific measurement availability across follow-up. Each cell reports the available number of measurements, the outcome-specific baseline N, and the corresponding percentage. Percentages describe measurement availability rather than participant retention because participant identities across annual summaries were not available in the published aggregate data used for this analysis. Values above 100% indicate that more measurements were reported at that follow-up point than at baseline.

### 3.2 Auxological patterns

The published mean height SDS was −2.38 (SD 0.63; n=318) at baseline and −0.76 (SD 0.96; n=42) at year 5, an observed difference of +1.62 SDS. The published mean weight SDS was −2.21 (SD 0.99; n=311) at baseline and −0.84 (SD 1.33; n=42) at year 5, an observed difference of +1.37 SDS. Mean BMI SDS was −0.93 (SD 1.04; n=311) at baseline and −0.64 (SD 1.42; n=42) at year 5, an observed difference of +0.29 SDS. These comparisons describe published annual group means rather than within-child changes. **Figure 2** presents the published annual aggregate means with pointwise 95% confidence intervals.

**Figure 2:**
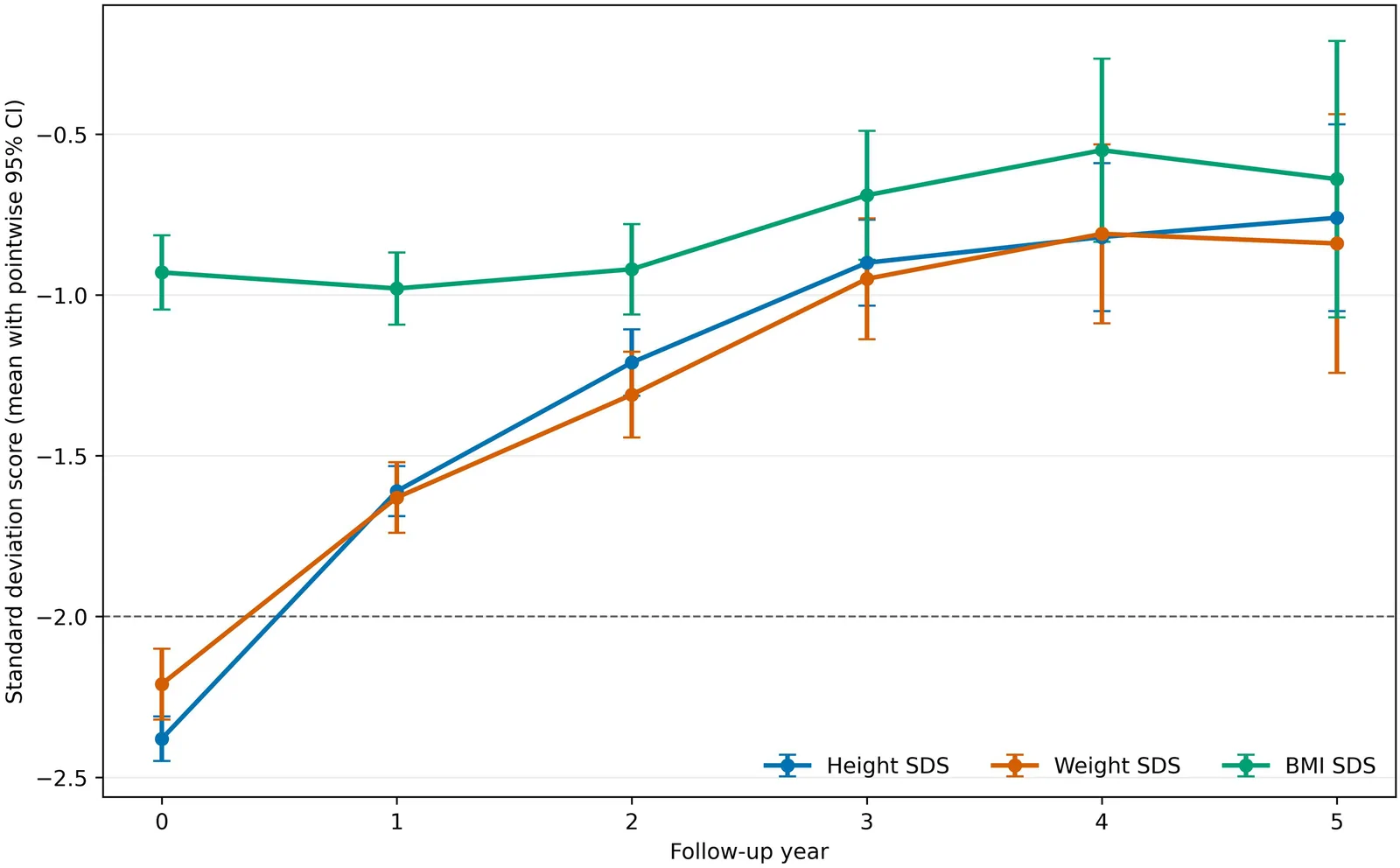
Annual auxological means reported for the SGA subgroup. Points represent published annual aggregate means. Error bars are pointwise 95% confidence intervals calculated as mean ± 1.96 (SD/√n). The annual means may represent changing participant subsets; connecting lines are descriptive and do not represent within-child trajectories.

### 3.3 Descriptive patterns across annual follow-up

**Table 2** presents the published annual mean, standard deviation, and outcome-specific sample size for each outcome from baseline through year 5, together with the observed year-5 minus baseline difference. These descriptive values form the primary empirical results.

**Table 2:** Published annual aggregate values and outcome-specific sample sizes.

Outcome	Baseline	Year 1	Year 2	Year 3	Year 4	Year 5	Observed Y5–baseline difference
Height SDS	−2.38 (0.63); n=318	−1.61 (0.66); n=276	−1.21 (0.72); n=187	−0.90 (0.75); n=121	−0.82 (1.05); n=80	−0.76 (0.96); n=42	+1.62 SDS
Weight SDS	−2.21 (0.99); n=311	−1.63 (0.93); n=275	−1.31 (0.93); n=187	−0.95 (1.05); n=120	−0.81 (1.27); n=80	−0.84 (1.33); n=42	+1.37 SDS
BMI SDS	−0.93 (1.04); n=311	−0.98 (0.95); n=275	−0.92 (0.98); n=187	−0.69 (1.12); n=120	−0.55 (1.30); n=80	−0.64 (1.42); n=42	+0.29 SDS
AST, IU/L	32.07 (7.53); n=307	28.78 (6.07); n=235	27.34 (5.74); n=151	26.49 (7.11); n=102	25.57 (5.80); n=60	24.28 (5.37); n=39	−7.79
ALT, IU/L	15.07 (6.26); n=307	14.34 (6.90); n=235	14.28 (6.63); n=151	14.67 (7.84); n=102	15.25 (6.66); n=60	15.46 (6.70); n=39	+0.39
Uric acid, mg/dL	3.92 (0.84); n=208	4.16 (0.89); n=153	4.32 (1.03); n=104	4.36 (0.74); n=62	4.60 (1.37); n=32	4.52 (1.34); n=19	+0.60
Total cholesterol, mg/dL	169.42 (27.45); n=273	164.36 (25.84); n=216	162.63 (23.85); n=137	164.94 (22.95); n=85	168.05 (27.87); n=46	159.69 (20.98); n=29	−9.73
Triglycerides, mg/dL	88.59 (57.73); n=70	92.44 (57.58); n=62	105.11 (58.90); n=28	120.00 (89.18); n=17	105.50 (63.32); n=8	107.50 (49.75); n=6	+18.91
Glucose, mg/dL	92.64 (13.49); n=296	97.03 (12.44); n=229	98.71 (10.92); n=149	97.07 (12.89); n=99	96.92 (10.72); n=60	100.53 (16.33); n=38	+7.89
HbA1c, %	5.29 (0.22); n=77	5.29 (0.27); n=136	5.32 (0.24); n=90	5.31 (0.26); n=70	5.36 (0.26); n=46	5.37 (0.26); n=29	+0.08

Values are published aggregate means (SD) with the outcome-specific number of reported measurements. Sample sizes differ across outcomes and years and do not define a common longitudinal cohort. Differences from baseline compare published annual group means and should not be interpreted as within-child changes or treatment effects.

The annual series did not support an assumption of uniform change across follow-up. The concentration of the largest height-SDS difference in the first follow-up interval further illustrates why a single linear slope would not adequately represent the shape of the published annual series. Height SDS changed by +0.77, +0.40, +0.31, +0.08, and +0.06 SDS across successive yearly intervals from baseline to year 5. Weight SDS showed changes of +0.58, +0.32, +0.36, +0.14, and −0.03 SDS over the same intervals. BMI SDS and several metabolic measures also fluctuated rather than following a constant linear trajectory. Accordingly, the main analysis describes differences among the published annual means rather than interpreting a single fitted slope as a uniform longitudinal treatment response. Across the metabolic outcomes, the published annual means were non-uniform. AST means were lower at later follow-up points, ALT means remained broadly similar, uric-acid means were generally higher, and total-cholesterol means fluctuated. Triglyceride values were particularly difficult to interpret because only six measurements were available at year 5. Glucose and HbA1c also showed modest variation across annual summaries. These comparisons describe published group means and do not estimate within-child biochemical change.

**Figure 3** expresses each metabolic outcome as the difference between each published annual mean and its baseline mean, standardized by the baseline SD. The display shows lower AST means at later follow-up points, generally higher uric-acid means, variable total-cholesterol means, and smaller differences for triglycerides, glucose, HbA1c, and ALT. Because cross-year covariance was not available in the published aggregate summaries used for this analysis, the heatmap is descriptive only.

**Figure 3:**
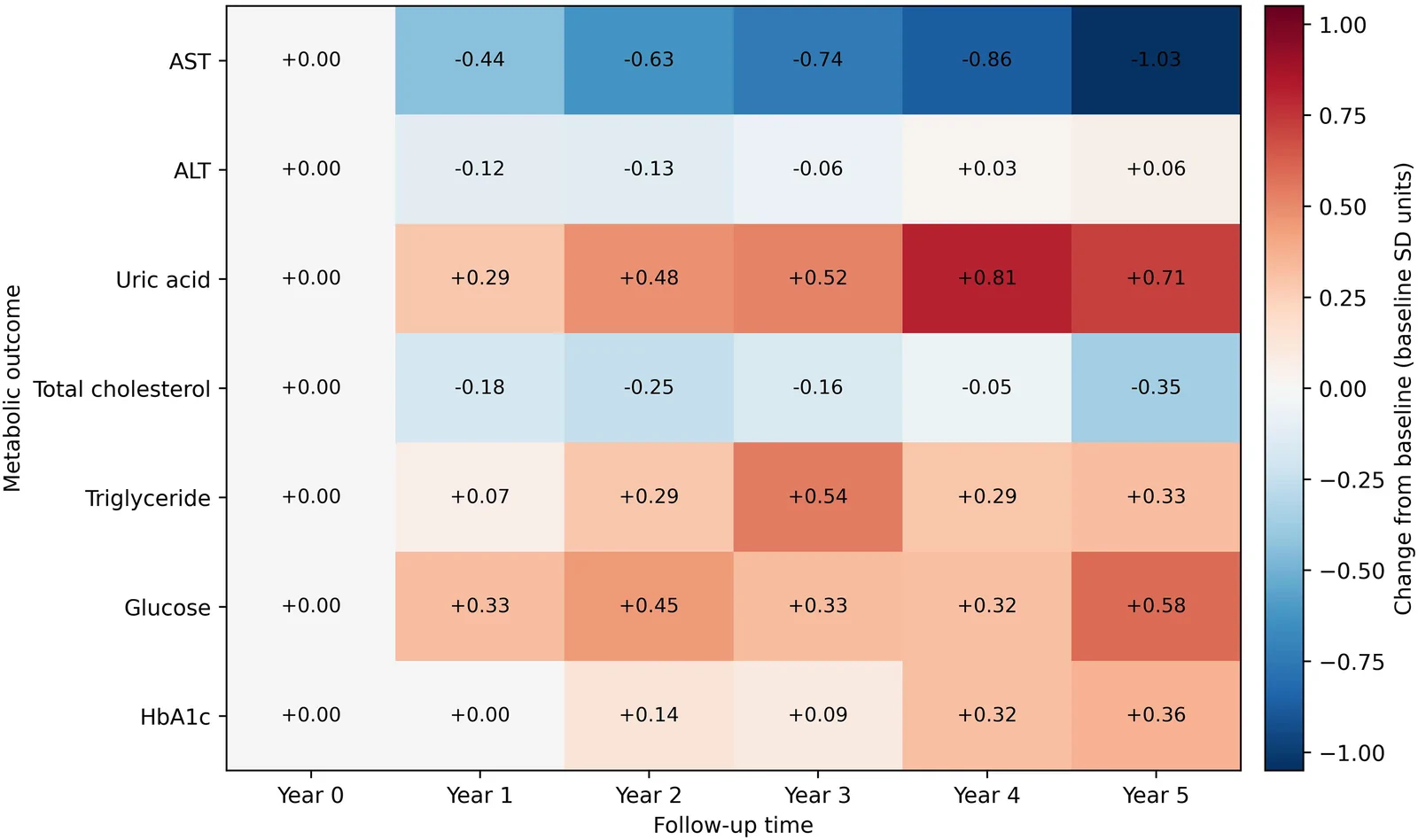
Differences in published annual metabolic means from baseline, expressed in baseline-SD units. Values are differences between each published annual aggregate mean and its outcome-specific baseline mean, divided by the baseline SD. The display supports directional comparison and does not represent a within-person standardized effect.

## 4 Discussion, contributions, and implications

### 4.1 Principal findings

The empirical results in this study are comparisons among published annual group means. They do not estimate within-child longitudinal change, treatment-associated effects, or causal effects. Age, sex, pubertal status, growth hormone dose, adherence, BMI, prematurity, comorbidities, family history, and baseline metabolic risk could not be adjusted for because individual-level data were not available to the present secondary analysis. Biological mechanisms are therefore discussed only as contextual hypotheses and not as explanations demonstrated by the present analysis.

The published annual summaries showed higher mean height and weight SDS at later follow-up points, a smaller difference in BMI SDS, lower AST means, and non-uniform patterns in the remaining metabolic measures. Late values were based on substantially smaller outcome-specific samples, particularly for uric acid and triglycerides. These observations therefore describe the direction of published annual group means rather than individual treatment responses.

The most important evidence boundary concerns cardiovascular outcomes. The primary aggregate data cannot determine whether GH exposure was related to hypertension, stroke, ischemic disease, or mortality. Favorable average glucose or cholesterol values cannot substitute for event surveillance, and the absence of cardiovascular events from a published summary table cannot be interpreted as evidence that no events occurred.

### 4.2 Interpretation in relation to prior evidence

The higher published mean height SDS values at later follow-up points are consistent with the broader efficacy literature on growth hormone treatment in children born SGA (5, 6, 8, 10, 16). However, the later annual means were based on fewer and potentially different participants, so the observed differences should not be translated into an expected within-child treatment response. Prior patient-level studies have reported more favorable growth outcomes with earlier treatment initiation, but the present aggregate analysis does not independently evaluate an initiation effect (6, 13).

BMI SDS showed a substantially smaller difference across the published annual means than height or weight SDS. It also aligns with studies reporting early BMI SDS reductions or favorable body-composition change (18, 29). Yet BMI alone cannot exclude central adiposity, and the published aggregate summaries used in the present analysis did not include waist circumference, DXA, MRI fat, or lean-mass series. A future registry should not use BMI as the sole metabolic phenotype.

Cardiovascular outcomes were outside the empirical scope of the primary dataset. The published aggregate summaries available for the present analysis did not include longitudinal blood-pressure series, cardiovascular-event records, person-time, or mortality linkage. Selected long-term studies are cited here only to provide clinical context: population-linkage studies have reported relative cardiovascular or mortality signals, whereas controlled post-treatment cohorts have not shown a clear treatment-specific adverse phenotype. Because these studies were not identified through a systematic or scoping-review process, the present discussion should not be interpreted as a comprehensive or unbiased synthesis of cardiovascular safety evidence.

The published mean HbA1c changed from 5.29% at baseline to 5.37% at year 5, an absolute difference of 0.08 percentage points. This small aggregate difference cannot establish incident diabetes, individual glycemic deterioration, or a treatment effect. Patient-level fasting glucose, insulin, HOMA-IR, pubertal status, BMI, family history, and medication data would be required to identify clinically meaningful risk.

Triglyceride interpretation is particularly limited by measurement availability. The published mean was 88.59 mg/dL at baseline and 107.50 mg/dL at year 5, but the latter value was based on only six measurements and standardized fasting information was not available in the published aggregate summaries used for this analysis. The appropriate interpretation is therefore that the published annual means differed, not that growth hormone caused dyslipidemia. Total-cholesterol means also varied across follow-up and should be interpreted descriptively. The published AST means were lower at later follow-up points, whereas ALT means changed little overall; however, aminotransferases are nonspecific biochemical measures.

### 4.3 Methodological contribution

The methodological contribution is to demonstrate how published annual registry summaries can be interpreted descriptively when participant-level covariance and follow-up identity are not available in the published summaries used for analysis. Presenting all annual means, standard deviations, and outcome-specific sample sizes makes measurement sparsity explicit while reducing the risk that group-level differences are mistaken for within-child change. Exploratory slope analyses are therefore treated only as supplementary sensitivity summaries.

### 4.4 Clinical and practical implications

The findings support careful interpretation of routine growth and metabolic monitoring rather than any change in treatment recommendations. Height, weight, and BMI should be considered jointly, while glycemic and lipid assessments should document fasting status where relevant. Future registry-based analyses would benefit from access to documented reasons for missing measurements and selected post-treatment follow-up, so that late outcome summaries are less vulnerable to selective measurement.

### 4.5 Implications for registry development in China

The same data-quality challenges are relevant to emerging Chinese growth-hormone registries, particularly incomplete late follow-up and non-standardized metabolic and blood-pressure measurement (27, 28). However, the analyzed cohort was Korean, and none of the numerical estimates in this study should be interpreted as outcomes for Chinese patients.

## 5 Conclusion, limitations, and future research

### 5.1 Conclusion

Published annual mean values were higher for height and weight SDS at later follow-up points, while metabolic measures showed smaller and non-uniform differences across years. These observations describe changing annual groups rather than within-child trajectories. Outcome-specific measurement availability declined markedly, with especially sparse late data for uric acid and triglycerides. The published aggregate summaries available for the present secondary analysis did not include longitudinal blood-pressure measurements, cardiovascular-event records, person-time, or mortality linkage. These summaries therefore cannot support estimation of causal treatment effects or cardiovascular risk. Exploratory slope analyses reported in the **Supplementary Material** yielded no statistically significant trend after false-discovery-rate correction (all FDR q ≥.0816).

### 5.2 Limitations

The limitations described below primarily reflect the published aggregate data available for the present secondary analysis. Because the authors did not have access to the original individual-level LGS database, absence of information from the analyzed summaries should not be interpreted as evidence that the corresponding variables or longitudinal information were not collected in the underlying LGS registry.

A fundamental limitation of the data available for the present secondary analysis is that the published annual means may represent overlapping but nonidentical participant subsets, while cross-year covariance was not available in the published aggregate summaries. Differences between annual group means therefore cannot be interpreted as individual longitudinal changes. The exploratory slope analyses reported in the **Supplementary Material** cannot overcome this limitation because individual trajectories and dependence between annual summaries were not available to the present analysis.

Outcome availability declined substantially and differed by laboratory. Later means may be influenced by selective testing or follow-up. The published aggregate summaries do not provide sufficient information to determine whether missingness relates to response, adverse effects, treatment cessation, cost, puberty, or clinician preference. No imputation method applied to these aggregate summaries can resolve this uncertainty without individual records and predictors of missingness.

The published aggregate summaries used for this analysis did not provide consistent fasting status, insulin or HOMA-IR trajectories, LDL or HDL cholesterol, blood pressure, body-composition imaging, medication data, or threshold-based clinical outcomes. This statement describes the information available for the present secondary analysis and does not establish that these variables were absent from the underlying LGS registry. Follow-up year was used as the only time metric because individual-level treatment-exposure data were not available to the present analysis. The available aggregate data did not permit adjustment for dose, adherence, puberty, sex, gestational age, prematurity, family history, or center. Because the analyzed cohort was Korean, the results should not be interpreted as estimates for Chinese patients. The literature synthesis emphasized contemporary evidence and may therefore underrepresent some historical primary studies.

### 5.3 Future research

Future research addressing these questions will require access to patient-level longitudinal data containing growth-hormone dose, adherence, puberty, adiposity, standardized fasting status, and cardiovascular outcomes, ideally with untreated SGA comparators and post-cessation follow-up. Such data would permit mixed-effects and time-to-event analyses that cannot be justified from the published aggregate summaries used here.
