# Supplementary material for: Exploratory secondary analysis of published aggregate growth and metabolic data in children born small for gestational age treated with growth hormone
Source: Front Endocrinol (Lausanne). 2026 Sep 25;17:1942946. doi: 10.3389/fendo.2026.1942946 (PMC13650744; doi:10.3389/fendo.2026.1942946)
Supplement: Supplementary file 1 [file SupplementaryFile1.docx]

**SUPPLEMENTARY MATERIAL**

**Exploratory Secondary Analysis of Published Aggregate Growth and Metabolic Data in Children Born Small for Gestational Age Treated With Growth Hormone**

This supplementary file reports exploratory aggregate-level slope models and sensitivity analyses based on the published annual means, standard deviations, and outcome-specific sample sizes used in the main manuscript. The analytical unit is the annual outcome summary rather than the individual child.

# Supplementary Methods

Exploratory aggregate-level slope models. For each of ten outcomes, a linear model was fitted to the six published annual aggregate means (baseline through year 5) using inverse-variance weights defined as N/SD². HC3 heteroskedasticity-robust standard errors were used, and confidence intervals and p values were calculated with a Student t reference based on four residual degrees of freedom. Benjamini-Hochberg false-discovery-rate correction was applied across the ten exploratory outcome models. Because annual samples may overlap and cross-year covariance is unavailable, these models are sensitivity summaries only; they do not constitute repeated-measures inference or estimates of within-child or causal treatment effects.

Sensitivity analyses. Three additional specifications were evaluated: an inverse-variance weighted model restricted to years 0-3, an unweighted OLS model with HC3 covariance, and leave-one-time-point-out analyses in which each annual summary was omitted in turn. Table S2 records only whether the fitted aggregate slope had the same sign across the evaluated specifications. This sign comparison is descriptive and does not establish statistical robustness, within-child change, causality, or clinical significance.

# Supplementary Table S1

*Exploratory aggregate-level slope model results.*

| **Outcome** | **Unit** | **Slope/year** | **HC3 SE** | **95% CI** | **p value** | **FDR q** | **Std. slope/year** |
| --- | --- | --- | --- | --- | --- | --- | --- |
| Height SDS | SDS | 0.430 | 0.164 | -0.025 to 0.886 | .0586 | .0977 | 0.683 |
| Weight SDS | SDS | 0.354 | 0.098 | 0.083 to 0.626 | .0222 | .0816 | 0.358 |
| BMI SDS | SDS | 0.076 | 0.052 | -0.070 to 0.221 | .2217 | .3167 | 0.073 |
| AST | IU/L | -1.537 | 0.482 | -2.875 to -0.199 | .0332 | .0816 | -0.204 |
| ALT | IU/L | 0.002 | 0.300 | -0.830 to 0.835 | .9940 | .9940 | 0.000 |
| Uric acid | mg/dL | 0.151 | 0.047 | 0.020 to 0.281 | .0325 | .0816 | 0.180 |
| Total cholesterol | mg/dL | -1.359 | 1.237 | -4.794 to 2.077 | .3339 | .3902 | -0.049 |
| Triglyceride | mg/dL | 5.421 | 1.820 | 0.367 to 10.475 | .0408 | .0816 | 0.094 |
| Glucose | mg/dL | 1.264 | 1.199 | -2.065 to 4.594 | .3512 | .3902 | 0.094 |
| HbA1c | % | 0.0159 | 0.0046 | 0.0030 to 0.0287 | .0266 | .0816 | 0.072 |

Note. Slopes are linear fits to published annual aggregate means and do not represent within-patient treatment effects. Confidence intervals and p values use HC3 standard errors with a Student t(4) reference. Because annual samples may overlap and their covariance is unknown, these quantities are exploratory summary-level measures. FDR q values were calculated across ten outcomes. No outcome reached q<.05; the minimum FDR q value was .0816. Std. slope/year = slope divided by the outcome-specific baseline SD.

# Supplementary Table S2

Sensitivity analyses of aggregate slopes.

| **Outcome** | **Weighted slope, years 0-5** | **Weighted slope, years 0-3** | **Unweighted slope** | **Leave-one-time-point-out range** | **Direction** | **Interpretation** |
| --- | --- | --- | --- | --- | --- | --- |
| Height SDS | 0.430 | 0.527 | 0.308 | 0.276 to 0.476 | Positive in all evaluated specifications | Positive sign in all evaluated aggregate specifications |
| Weight SDS | 0.354 | 0.425 | 0.276 | 0.266 to 0.389 | Positive in all evaluated specifications | Positive sign in all evaluated aggregate specifications |
| BMI SDS | 0.076 | 0.055 | 0.085 | 0.059 to 0.123 | Positive in all evaluated specifications | Positive sign in all evaluated specifications; magnitude small |
| AST | -1.537 | -1.988 | -1.412 | -1.679 to -1.097 | Negative in all evaluated specifications | Negative sign in all evaluated aggregate specifications |
| ALT | 0.002 | -0.250 | 0.145 | -0.077 to 0.283 | Varied across specifications | No common sign across evaluated specifications |
| Uric acid | 0.151 | 0.158 | 0.125 | 0.108 to 0.167 | Positive in all evaluated specifications | Positive sign in all evaluated aggregate specifications |
| Total cholesterol | -1.359 | -1.933 | -1.008 | -1.786 to -0.247 | Negative in all evaluated specifications | Negative sign in all evaluated specifications; magnitude uncertain |
| Triglyceride | 5.421 | 8.717 | 4.246 | 4.703 to 6.937 | Positive in all evaluated specifications | Positive sign in all evaluated specifications; year-5 data extremely sparse (n=6) |
| Glucose | 1.264 | 1.973 | 1.071 | 0.192 to 1.747 | Positive in all evaluated specifications | Positive sign in all evaluated specifications; estimate imprecise |
| HbA1c | 0.0159 | 0.0099 | 0.0171 | 0.0139 to 0.0191 | Positive in all evaluated specifications | Positive sign in all evaluated specifications; absolute difference small and not diagnostic |

Note. Direction labels record only whether the fitted aggregate slope had the same sign across the evaluated specifications. They do not demonstrate statistical robustness, causality, within-person change, or clinical significance. The triglyceride analysis is especially limited by only six reported measurements at year 5.

# Supplementary Figure S1

Supplementary Figure S1. Standardized exploratory slopes fitted to published annual aggregate means.

Note. Points are inverse-variance weighted aggregate slopes divided by the corresponding baseline SD; bars are HC3-based 95% confidence intervals using a Student t(4) reference. All outcomes use the same visual treatment, and nominal p<.05 findings are not highlighted. No outcome remained statistically significant after Benjamini-Hochberg false-discovery-rate correction (all q≥.0816). The figure is an exploratory sensitivity display and does not represent within-child longitudinal change.

# Supplementary Reproducibility Statement

The supplementary calculations were generated from the same public aggregate workbook used for the main manuscript, containing 60 outcome-year records (10 outcomes × 6 annual time points). Before model fitting, the workflow checked numeric input fields, positive standard deviations, sample sizes greater than one, unique outcome-year records, and availability of years 0-5 for each outcome. The minimum multiplicity-adjusted q value across the ten exploratory slopes was .0816.
